# Lightweight precise automatic extraction of exception preconditions in java methods

**DOI:** 10.1007/s10664-023-10392-x

**Published:** 2023-12-26

**Authors:** Diego Marcilio, Carlo A. Furia

**Affiliations:** Software Institute, USI Università della Svizzera italiana, Lugano, Switzerland

**Keywords:** Java, Java exceptions, Preconditions

## Abstract

When a method throws an exception— its *exception precondition*—is a crucial element of the method’s documentation that clients should know to properly use it. Unfortunately, exceptional behavior is often poorly documented, and sensitive to changes in a project’s implementation details that can be onerous to keep synchronized with the documentation. We present wit, an automated technique that extracts the exception preconditions of Java methods and constructors. wit uses static analysis to analyze the paths in a method’s implementation that lead to throwing an exception. wit’s analysis is precise, in that it only reports exception preconditions that are correct and correspond to feasible exceptional behavior. It is also lightweight: it only needs the source code of the class (or classes) to be analyzed— without building or running the whole project. To this end, its design uses heuristics that give up some completeness (wit cannot infer all exception preconditions) in exchange for precision and ease of applicability. We ran wit on the JDK and 46 Java projects, where it discovered 30 487 exception preconditions in 24 461 methods, taking less than two seconds per analyzed public method on average. A manual analysis of a significant sample of these exception preconditions confirmed that wit is 100% precise, and demonstrated that it can document the exceptional behavior of Java methods.

## Introduction

To correctly use a method, we must know its *precondition*, which specifies the *valid* inputs: those that the method’s implementation can handle correctly. In programming languages like Java, a method’s implementation may throw an *exception* to signal that a call violates its precondition. If it does so, knowing the method’s exceptional behavior is equivalent to knowing (the complement of) its precondition. Ideally, a method’s exceptional behavior should be described in the method’s documentation (for example, in its Javadoc comments) and thoroughly tested. In practice, it is known that a method’s documentation can be incomplete or inconsistent with its implementation Nassif et al. (2021); Zhou et al. (2020), and that only a fraction of a project’s test suite exercises exceptional behavior Marcilio and Furia (2021). This ultimately limits the usability, in a broad sense, of insufficiently documented methods: without precisely knowing its precondition, programmers may have a hard time calling a method; test-case generation may generate invalid tests that violate the method’s precondition; program analysis may have to explicitly follow the implementation of every called method, which does not scale since it is not modular.

To alleviate these problems, we present wit (*What Is Thrown?*): a technique to automatically infer the *exception preconditions*—the input conditions under which an exception is thrown—of Java methods. As we discuss in Section 7, extracting preconditions and other kinds of specification from implementations is a broadly studied problem in software engineering (and, more generally, computer science). Our wit approach is novel because it offers a distinct combination of features. First, wit is *precise*: since it is based on static analysis, it reports preconditions only when it can determine with certainty that they are correct. It is also *lightweight*, as it is applicable to the source code of individual classes of a large project without requiring to build the project (or even to have access to all project dependencies), and can combine its analysis of multiple projects in a modular fashion.

A key assumption underlying wit’s design is that a significant fraction of a method’s exceptional executions are usually simpler, shorter, and easier to identify than the other, normal, executions. Therefore, wit’s analysis (which we describe in detail in Section 3) relies on several heuristics that drastically limit the depth and complexity of the program paths it explores—for example, it bounds the length of paths and number of calls that it can follow. Whenever a heuristics fails, wit gives up analyzing a certain path for exceptional behavior. In general, this limits the number of exception preconditions that wit can reliably discover. However, if our underlying assumption holds, wit can still be useful and effective, as well as lightweight and scalable.

We implemented wit in a tool with the same name, which performs a lightweight static analysis of Java classes using JavaParser for parsing and the Z3 SMT solver for checking which program paths are feasible. Section 4 describes an experimental evaluation where we applied wit to several modules of Java 11’s JDK, and 46 Java projects—including several widely used libraries—to discover the exception preconditions of their public methods. wit inferred 30 487 exception preconditions of 24 461 methods—running for 1.9 seconds on average on each of the 460 032 analyzed public methods.

A manual analysis of a significant random sample of the inferred preconditions confirmed that wit is precise: all manually checked preconditions were correct. It also revealed that it could retrieve 9–83%^1^ of all supported exception preconditions in project JApache Commons IO—achieving even higher recall on projects that use few currently unsupported Java features. Our empirical evaluation also indicates that wit can be *useful* to programmers: 38% of the exception preconditions in the JDK’s sample and 72% in the other projects’ were not already properly documented; and 7 pull requests—extending the public documentation of open-source projects with a selection of wit-inferred preconditions—were accepted by the projects’ maintainers.

### Contributions

In summary, the paper makes the following contributions:

- wit: a technique to automatically infer the exception preconditions of Java methods based on a novel combination of static analysis and heuristics that trade-off exhaustiveness for high precision.
- An implementation of wit and an experimental evaluation targeting five JDK 11 modules and 46 open-source Java projects (including popular ones like Apache JCommons Lang, and the Jh2database), which demonstrates wit’s effectiveness, practical applicability to real-world projects, and usefulness.
- For reproducibility, wit’s implementation and the detailed experimental outputs are available.^,^^2^

### Extended Version

This article extends our previous work *What Is Thrown? Lightweight Precise Automatic Extraction of Exception Preconditions in Java Methods*, published at the ICSME 2022 conference Marcilio and Furia (2022) with improvements to the wit technique and its implementation, as well as a substantial extension to the experimental evaluation, which now includes a significant fraction of Java 11’s JDK libraries. Correspondingly, the experimental evaluation also explicitly investigates the impact of one of wit’s new features: modular analysis (introduced in Section 3.4).

## Showcase Examples of Using wit

We briefly present examples of applying wit to detect the exception preconditions of library functions in two Apache projects: Dubbo^3^ and Commons Lang.^4^ The examples showcase wit’s capabilities and practical usefulness: wit could automatically extract exception preconditions in many methods of these two projects, including some that were not documented (Section 2.1) or incorrectly documented (Section 2.2). Section 5.6 reports further empirical evidence that wit’s exception preconditions can be useful as a source of documentation.

To better gauge wit’s capabilities, let us stress that the two Apache projects discussed in this section are widely used Java libraries; for instance, Dubbo’s GitHub repository^5^ has over 24 thousand forks and 36 thousand stars. As a result, they are particularly well documented and tested Zhong et al. (2020); Nassif et al. (2021). The fact that wit could find some of their few missing or inconsistent pieces of their documentation indicates that it has the potential to be practically useful and widely applicable.
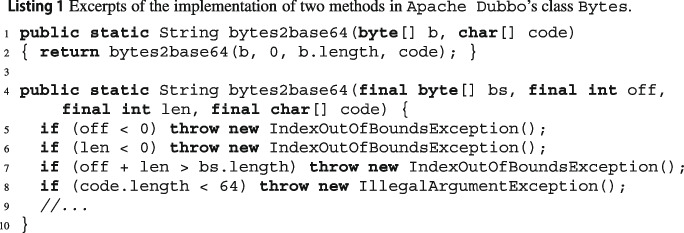


### Missing Documentation

Listing 1 shows an excerpt of two overloaded implementations of method Jbytes2base64, which takes a byte array and represents it as a string in base 64. As we can see from the initial lines in Jbytes2base64’s second implementation, the two methods have fairly detailed preconditions; furthermore, since the first method calls the second with additional fixed argument values, the first’s precondition is a special case of the second’s. Unfortunately, the documentation of these methods does not mention these preconditions: for example, the second method’s Javadoc comment vaguely describes Joff and Jlen as simply “offset” and “length”, without clarifying that they should be non-negative values. This lack of documentation about valid inputs decreases the usability of the methods for users of the library.

Running wit on class JBytes automatically finds the preconditions of these (as well as many other) methods, thus providing a useful form of rigorous documentation. For instance, one of the exception preconditions found by wit for Listing 1’s second method:

**Table:** 

*throws:*	JIndexOutOfBoundException
*when:*	Joff>= 0 && len>= 0 && bs.length< len + off
*example:*	J[off=0, len=1, bs.length=0]

corresponds to the path that reaches line 7 in Listing 1. wit also understands that the first method never throws this exception, but it can still throw others such as:

**Table:** 

*throws:*	JIllegalArgumentException
*when:*	Jb.length>= 0 && code.length< 64
*example:*	J[b.length=0, code.length=0]

In fact, wit only reports exception preconditions that correspond to *feasible* paths. Each precondition comes with an example of argument values that make the precondition true. These are not directly usable as test inputs, since they describe the input’s properties without constructing them; but they are useful complements to the precondition expressions, and help users get a concrete idea of the exceptional behavior.
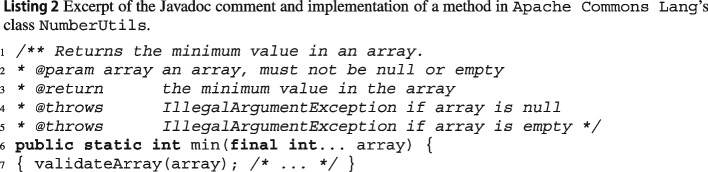


### Inconsistent Documentation

Listing 2 shows the complete Javadoc documentation and a brief excerpt of method Jmin in the latest version of Apache Commons Lang’s class JNumberUtils, which computes the minimum of an Jarray of integers. Unlike the previous example, Jmin’s documentation is detailed and clearly expresses the conditions under which an exception is thrown. Unfortunately, the documentation is partially incorrect: when Jarray is null, Jmin throws a JNullPointerException, not an JIllegalArgumentException, as precisely reported by wit:

**Table:** 

*throws:* JNullPointerException *when:* Jarray == null	

This inconsistency is due to a change in the implementation of JvalidateArray, which is called by Jmin to validate its input and uses methods of class JValidate to perform the validation. In version 3.12.0 of the library, JvalidateArray switched^6^ from calling JValidate.isTrue(a!=null) (which throws an JIllegalArgumentException when the check fails) to calling JValidate.notNull(a) (which throws a JNullPointerException instead) to check that Ja is not null.

To help locate the source of any exceptional behavior, wit also outputs the line where the exception is thrown, and possibly the triggering method call. In this example, it would clearly indicate that the exceptional behavior comes from a call to JValidate.notNull.

This information can help detect and debug such inconsistencies, which would be quite valuable to project developers and users. As we discuss in Section 5.6, maintainers of Apache libraries were appreciative of our pull requests which extended the projects’ documentation with some of wit’s exception preconditions.

## How wit Works

**Fig. 1:**
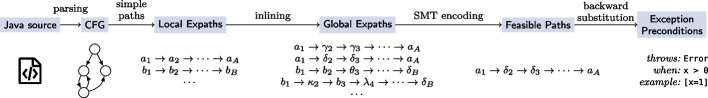
An overview of how wit works. wit parses the source code of the Java classes to be analyzed, and builds a control-flow graph (CFG) of every method. It enumerates the simple paths in every method’s CFG that may end with an exception (expaths). It then transforms these expaths local to a specific method into global expaths by inlining method calls or previously extracted exception preconditions (if they are available); this may transform a single local expath into multiple global expaths. To determine which expaths are feasible, wit encodes their constraints as an SMT problem and uses the Z3 SMT solver to check if they are satisfiable. It finally transforms all feasible paths into *exception preconditions*

Figure 1 overviews how wit’s analysis works.

This section details each step and discusses some features of its current implementation.

wit inputs the source code of some Java classes; it analyzes the methods and constructors of those classes to determine their *exception preconditions*, that is the conditions on the methods’ input that lead to the methods throwing an exception. It then outputs the exception preconditions it could find, together with their matching exception class, as well as examples of inputs that satisfy the exception preconditions. wit’s analysis only needs the source code of the immediate classes to be analyzed: it does not need a complete project’s source code, nor to compile or build the project.

wit can analyze both regular methods and constructors of a class. Thus, for brevity, we use the term “methods” to collectively refer to both methods and constructors.

### Parsing and CFG

wit parses the source code given as input using JavaParser,^7^ and constructs a control-flow graph (CFG) of the methods in the input classes using library JGraphT.^8^ More precisely, we build a CFG for each method Jm individually; and annotate branches in the CFG with each branch’s Boolean condition.

Listing 3 shows excerpts of 3 methods of class JArrayUtils^9^ in Apache Commons Lang. Method Jinsert puts some values Jv into an array Ja of Booleans at a given index Jk. The initial part of its implementation calls another method, JisEmpty, of the same class to determine if Jv is empty; in turn, JisEmpty calls method JgetLength. wit builds CFGs for Jinsert, JisEmpty, and JgetLength, since they are all part of the input source code.
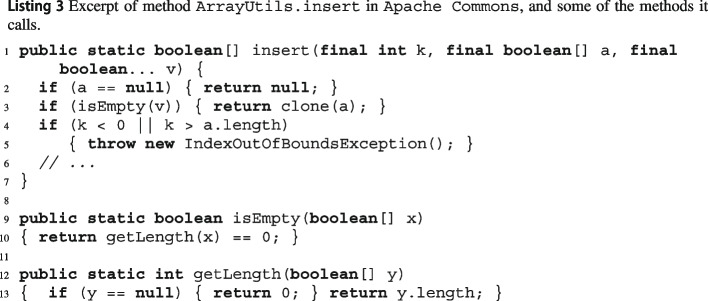


### Local Exception Paths

When analyzing a method Jm, wit collects its *local exception paths* (“expaths” for short). These are all simple directed paths^10^^,^^11^ on Jm’s CFG that end with a node corresponding to a statement that may throw an exception—either explicitly with a Jthrow or indirectly with a a *call* (which may return exceptionally).

In Listing 3’s example, one of Jinsert’s local expaths *p* goes through the else branch on lines 2–3 and through the *then* branch on line 4, ending with the Jthrow on line 5:

$$ p:J{if}_{2} \xrightarrow {J{a!=null}} J{if}_{3} \xrightarrow {J{!isEmpty(v)}} J{if}_{4} \xrightarrow {J{k<0 \Vert \Vert k>a.length}} J{throw}_{5} $$

### Global Exception Paths

After collecting expaths local to each method, wit converts them into *global* expaths by *inlining* calls to other methods.

Given a local expath $\ell$, for each node $n_{J{x}}$ in $\ell$ that calls some other method Jx, wit checks whether Jx’s CFG is available (that is, whether Jx’s implementation was part of the input). If it is, wit enumerates all simple paths that go through the CFG of Jx, and splices each of them into $\ell$ at $n_{J{x}}$. In other words, it transforms the local path $\ell$ so that it follows inter-method calls. Since a method usually has multiple paths, one local expath may determine several global expaths after inlining. wit inlines calls recursively (with some limits that we discuss in Section 3.7).

When a called method Jx’s CFG is not available in the current run, wit first looks whether it analyzed Jx’s source code in some of its previous runs. If this is the case, wit replaces the call to Jx with Jx’s exception preconditions it extracted in the previous runs —following the modular analysis procedure we explain in Section 3.4. Otherwise, if no information about Jx is available or the user deliberately disabled modular analysis, wit doesn’t inline calls to it and marks them as “opaque”.

wit inlines the call to JisEmpty in local expath *p* (Listing 3’s example) since JisEmpty is part of the same analyzed class JArrayUtils. Inlining the call replaces *p*’s edge $J{if}_{3} \xrightarrow {J{!isEmpty(v)}} J{if}_{4}$ with JgetLength’s only path: $J{if}_{3} \xrightarrow {J{!(getLength(v)==0)}} J{if}_{4}$. Since the implementation of JgetLength is available too, wit recursively inlines its two paths, which finally gives two global expaths $p_1, p_2$ that inline Jinsert’s local expath *p*’s calls:

$$\begin{aligned} p_1&:J{if}_{2} \rightarrow J{if}_{3} \rightarrow J{if}_{13} \xrightarrow {J{v==null},\, J{0 != 0}} J{if}_{4} \rightarrow J{throw}_{5} \\ p_2&:J{if}_{2} \rightarrow J{if}_{3} \rightarrow J{if}_{13} \xrightarrow {J{v!=null}, J{v.length != 0}} J{if}_{4} \rightarrow J{throw}_{5} \end{aligned}$$

### Modular Analysis

By default, wit saves all exception preconditions it extracts—together with their associated global exception paths—in a database, so that they can be reused to perform a modular analysis. This is useful whenever a method Jm in some project *A* calls another method Jn in some other project *B*. If we provide *A* and *B* in a single run, wit’s analysis has access to all the source code; thus, in principle, it can inline the code of *B*’s Jn when analyzing *A*’s Jm. However, this may not scale, as the number of paths to be considered grows like the product of Jm’s and Jn’s paths. To perform modular analysis, we instead first run wit on *B* alone; then, we run it on *A* alone. When wit analyzes Jm in *A*, it finds that it calls an external method Jn in *B*; thus, it reuses Jn’s saved exception precondition information to analyze the exceptional behavior of Jm when analyzing *A* without having to analyze Jn again (or without treating it like an opaque method, which may miss information).^12^
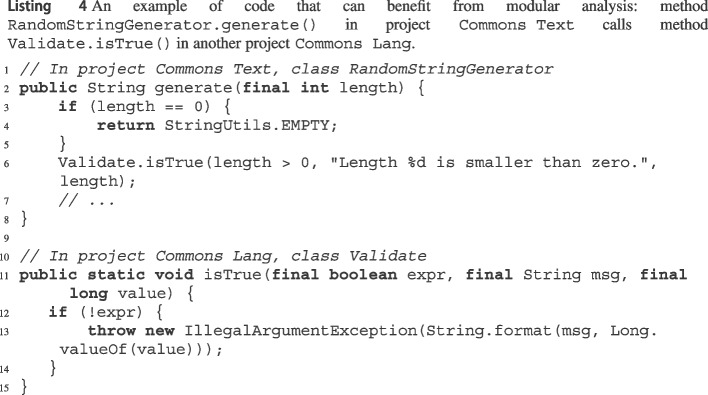


More precisely, if modular analysis is enabled, whenever a node $n_{J{x}}$ in a local expath $\ell$ calls a method Jx that was analyzed in a previous run, wit replaces the call to Jx by inlining any global exception path associated with Jx’s exception preconditions (and replacing, as usual, Jx’s formal parameters with the actual call arguments). Just like regular inlining (Section 3.3), this may introduce multiple global expaths for a single call to Jx. It is necessary, in general, to consider all available global expaths for a called method, so that all possible side effects of the call are accounted for. wit can use both expres and maybes for modular analysis.^13^ Since maybes are not guaranteed to be correct, any global expath that includes a maybe is automatically also classified as maybe.

As an example of where modular analysis can improve wit’s capabilities, consider Listing 4. Method Jgenerate^14^ of class RandomStringGenerator in project Commons Text calls method Validate.isTrue^15^ in another project Commons Lang. If we run wit on project Commons Text alone, the call to isTrue is marked as opaque, and hence no exception precondition would be reported for this path. We could run wit on both projects Commons Text and Commons Lang together; this would take a considerable amount of time, and it would not scale to combining even more projects. Instead, we can use wit’s modular analysis and first analyze Commons Lang in isolation; this would report the exception precondition !expr for method validate.isTrue. Then, when wit runs on Commons Text, it would replace the call to isTrue in generate with if (!(length> 0)) throw new IllegalArgumentException(), which leads to inferring exception precondition length $\mathtt {<= 0}$ for this path in method generate.

As we will demonstrate in Section 5, modular analysis can boost wit’s output and help achieve a better scalability. Implementation-wise, wit persists JSON objects into a MongoDB^16^ instance. For JSON serialization and deserialization, we combine JavaParser’s serialization package^17^ with the Moshi JSON library.^18^
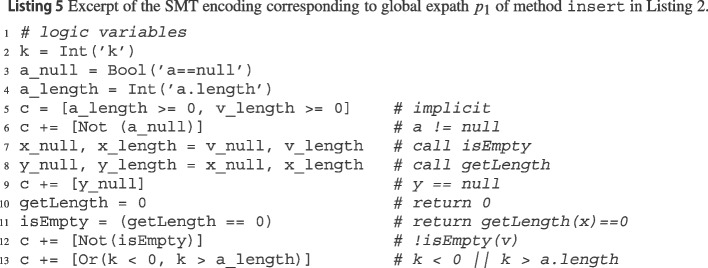


### Path Feasibility

wit builds global expaths only based on syntactic information in the CFGs; therefore, some paths may be infeasible (not executable). To determine whether a global expath is feasible, wit encodes it in logic form as an SMT (Satisfiability Modulo Theory) formula Barrett et al. (2009), and uses the Z3 SMT solver Mendonça de Moura and Bjørner (2008) to determine whether the expath’s induced constraints are feasible.

To this end, it first transforms the path into SSA (static single assignment) form, where complex statements are broken down into simpler steps, and fresh variables store the intermediate values of every expression. We designed a logic encoding of Java’s fundamental types (int, boolean, byte, arrays, strings) with their most common operations (including arithmetic, equality, length, contains, isEmpty), as well as of a few widely used JDK library methods (such as Array.getLength). wit uses this encoding to build an SMT formula $\phi$ corresponding to each global expath *p*: if $\phi$ is satisfiable, then the global expath *p* is feasible, and hence it corresponds to a possible exceptional behavior of method m.

wit encodes $\phi$ as a Python program using the Z3 SMT solver’s Z3Py Python API.^19^ Listing 5 shows a simplified excerpt of the SMT program encoding the feasibility of insert’s global expath $p_1$. First, it declares logic variables of the appropriate types to encode program variables (e.g., k), their basic properties (e.g., a_length, which corresponds to the Java expression a.length), and the values passed via method calls (e.g., getLength is an integer variable storing getLength()’s output). Then, it builds a list c of constraints that capture the path constraints and the semantics of the statements along the path. For example, a_length must be nonnegative, since it corresponds to array a’s length (line 5); the properties of array v are copied to those of x, since insert’s argument v is the actual argument for isEmpty’s formal argument x (line 7); and path constraint !isEmpty(v) corresponds to the complement of Boolean variable isEmpty (line 12). In this case, Z3 easily finds that the constraints in c are unsatisfiable, since Not(0 == 0) is identically false. In contrast, the constraints corresponding to path $p_2$ are satisfiable, and thus Z3 outputs a satisfying assignment of all variables in that case.

Sometimes wit does not have sufficient information to determine with certainty whether a path is feasible. When a path includes a call to an opaque method (whose implementation is not available or when the analysis fails) wit’s feasibility check is underconstrained. In these cases, wit still performs a feasibility check but reports any results as *maybe*, to warn that the output may not be correct.

In Listing 3’s example, suppose that getLength’s implementation wasn’t available.

In this scenario, based on its signature,

wit would only know that getLength returns an integer without any constraints; therefore it would classify path *p* as feasible but mark it as *maybe* since it is just an educated guess without correctness guarantees.

### Exception Preconditions

A feasible path *p* identifies a range of inputs of the analyzed method m that trigger an exception. In order to characterize those inputs as an *exception precondition*, wit encodes *p*’s constraints as a formula that only refers to m’s arguments, as well as to any members that are accessible at m’s entry (such as the target object this, if m is an instance method). To this end, it works backward from the last node of exception path *p*; it collects all path constraints along *p*, while replacing any reference to local variables with their definition. For example, method void f(int x){int y=x+1; if(y> 0)throw;} has a single feasible expath with path condition y> 0, which becomes x + 1> 0 after backward substitution through the assignment to variable y. Since x + 1> 0 only mentions argument x, it is a suitable exception precondition for method m.

Sometimes wit cannot build an exception precondition expression that only mentions arguments and other visible members. A common case is when a path includes opaque calls: since the semantics or implementation of these calls is not available, any expressions including them may not make sense in a precondition. In all these cases, wit still reports the exception expression obtained by backward substitution, but marks it as a *maybe* to indicate that it may not be correct. Another, more subtle case occurs when the exception precondition Boolean expression includes calls to methods (as opposed to just variable lookups). If these methods are not *pure* (that is, they do not change the program state), the precondition may be not well-formed. For instance, a precondition x.inc() == 0, where calling inc increments the value of x. Here too, wit is conservative and marks as *maybe* any exception precondition that involves calls to methods that are not known to be pure.

Before outputting any exception preconditions to the user, wit *simplifies* them to remove any redundancies and display them in a form that is easier to read. To this end, it uses SymPy Meurer et al. (2017),^20^ a Python library for symbolic mathematics. Java’s syntax is sufficiently similar to C’s that we can also enable SymPy’s pretty printing of expressions using C syntax, and then additionally tweak it to amend the remaining differences with Java. While conceptually simple, the simplification step is crucial to have readable exception preconditions. For example, SymPy simplifies the ugly expression 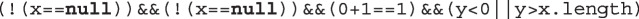 into the much more readable 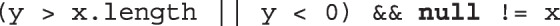, which doesn’t repeat 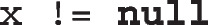 and omits the tautology 0 + 1 == 1.

wit’s final output consists of a series of tuples with: (a) an exception precondition, (b) whether it is a *maybe*, (c) the thrown exception type, (d) and an example of inputs that satisfy the precondition (given by Z3’s successful satisfiability check).

For debugging, wit can also optionally report the complete throw statement (including any exception message or other arguments used to instantiate the exception object), the line in the analyzed method m where the exception is thrown or propagated, and a sequence of method calls starting from the analyzed method and ending in the throwing method. Moreover, wit reports the generated Z3 and SymPy Python programs’ source code.

### Heuristics and Limitations

Let us now zoom in on a few details of how wit’s implementation works, which clarify its capabilities and limitations. To put these details into the right perspective, let us recall wit’s design goals: it should be precise and lightweight; it’s acceptable if achieving these qualities loses some generality— as long as a sizable fraction of exception preconditions can be precisely determined.

**Using maybes** As discussed in Section 3.5, wit provides two disjoint sets of exceptional preconditions as output: expres and maybes. In practice, reporting both gives users more flexibility in how to use wit’s output according to different use cases. If correctness is crucial (for example, if one uses wit’s output as formal specification), then users should only consider expres and ignore maybes. On the other hand, if some degree of uncertainty in the correctness of an exception precondition is acceptable in exchange for a higher recall, then users may also consider maybes. The snag is that they may have to spend extra effort to validate the maybes, but this may be acceptable if there exist practical validation means (for example, an extensive test suite). Any kind of hybrid approach is also possible; for instance, one may first only use expres, but consider using maybes selectively for a few methods where wit’s feasibility analysis struggled due to the features used there.

**Implicit exceptions** wit only tracks exceptions that are explicitly raised by a throw statement; it does not consider low-level errors—such as division by zero, out-of-bound array access, and buffer overflow—that are signaled by exceptions raised by the JVM. This restriction is customary in techniques that infer exceptional behavior, since implicitly thrown exceptions are “generally indicative of programming errors rather than design choices Weimer and Necula (2004)” Raymond and Weimer (2008), and usually do not belong in API-level documentation Forward and Lethbridge (2002) and are best analyzed separately. Extending wit to also track implicit exceptions would not be technically difficult; for example, one could first instrument the code to be analyzed with explicit checks before any statement that may thrown an implicit exception.^21^ However, indiscriminately considering all exceptions that are thrown implicitly would produce a vast number of boilerplate exception preconditions that are not specific to a method’s explicitly programmed behavior; hence, they would be outside wit’s current focus.

**Java features** wit’s CFG construction currently does not fully support some Java features: instanceof operators, for-each loops, switch statements, and try/catch blocks. When these features are used, the CFG may omit some paths that exist in the actual program. (Supporting the latter three features is possible in principle, but would substantially complicate the CFG construction.)^22^^,^^23^ The SMT encoding used for path feasibility (Section 3.5) is limited to a core subset of Java features and standard library methods. As a result, wit won’t report exception preconditions that involve unsupported features (or will report them as *maybe*, that is without correctness guarantee).

**Path length and number** In large methods, even some local expaths can be too complex, which bogs down the whole analysis process. Therefore, wit only enumerates paths of up to $N = 50$ nodes, which have a much higher likelihood of being manageable. Complex methods may have thousands of local paths. Therefore, wit analyzes up to $N = 500$ paths of a given method or constructor.

**Inlining limits** Inlining can easily lead to a combinatorial explosion in the number and length of the expaths; therefore, a number of heuristics limit inlining. First, a path can be inlined only if it is up to $N=50$ nodes—the same limit as for local expaths. Second, wit stops inlining a call in a path after it has reached a limit of $I=100$ inlined paths—that is, it has branched out the call into *I* different ways. It can still inline other calls in the same path, but this limit avoids recursive inlinings that are likely to blow up. Third, wit enumerates the inlinings of a call in random order; in cases where the limit *I* is reached, this increases the chance of collecting a more varied set of inlined paths instead of getting stuck in some particularly complex ones (if the limit *I* is not reached, the enumeration order is immaterial).

**Maybes heuristics** The feasibility of exception preconditions reported as maybes could not be verified; hence, they are educated guesses. Consequently, wit deploys two simple heuristics that filter out maybes that are overwhelmingly unlikely to be correct. First, wit does not report any maybe assertion that consists of more than six conjuncts or disjuncts; we found that the constraints of such large maybes are usually unsatisfiable. Second, wit drops any maybe that includes constraints over private fields of the JDK’s String and StringBuilder classes. This heuristic only applies when wit uses *modular* analysis: these two JDK classes have a complex implementation involving native code and JVM internals. Thus, wit’s analysis of String and StringBuilder can only retrieve a few correct maybes; as a result, using them in the modular analysis of other client classes is likely to introduce a large number of spurious maybes—which this heuristic avoids.

**Timeouts** Z3’s satisfiability checks (to determine if a path is feasible) may occasionally run for a long time. wit limits each call to Z3 to a $Z=15$-second timeout; when the timeout expires, Z3 is terminated and the path is assumed to be infeasible. There is also an overall timeout of $T=10$ minutes per analyzed class. If wit’s analysis still runs after the timeout, it probably means that the class’s methods are particularly intricate and hard to process;to remain lightweight, wit skips to the next class.

**Configurable options** The parameters regulating these heuristics can be easily changed if one needs to analyze code with peculiar characteristics, when a large running time is not a problem. wit also offers two slightly different Z3 logic encodings of some Java features. By default, it employs a conservative encoding that ensures that all expressions used in an exception precondition are well defined (for example, a.length implicitly requires that a != null). In some complex cases, this encoding may be overly conservative, leading to marking as unsatisfiable exception preconditions that are actually correct. To accommodate these unusual cases, wit also offers a less conservative logic encoding of the same features, which trades off correctness for recall; users can switch to this alternative encoding when analyzing software where a high recall is more important than an absolute correctness guarantee.

**Modular analysis** wit’s modular analysis (Section 3.4) is also configurable to fit each application scenario. By default, wit performs modular analysis: if it encounters a call to a method that it analyzed in a previous run, it uses the called method’s exception preconditions to determine the exception preconditions of the caller. In contrast, if the user explicitly disables modular analysis, wit analyzes each project in isolation. Section 5.4 describes experimental data that we collected to better understand the practical impact of using wit’s modular analysis. When modular analysis is enabled, wit can reuse only expres or both expres and maybes. This is another parameter that one can choose according to how important a high recall is: reusing also maybes can only increase the number of maybes inferred by wit, which come with no guarantee of being correct. In general, modular analysis is an additional option made available by wit, which need not be used in all situations: whether enabling it is beneficial depends on the projects under analysis and on the user’s requirements.

## Experimental Evaluation

This section describes the empirical evaluation of wit, which targets the following research questions.

1. (precision): How many of the exception preconditions detected by wit are correct?
2. (recall): How many exception preconditions can wit detect?
3. (features): What are the most common features of the exception preconditions detected by wit?
4. (modularity): How do the exception preconditions detected by wit change if modular analysis is disabled?
5. (efficiency): Is wit scalable and lightweight?
6. (usefulness): Are wit’s exception preconditions useful to complement programmer-written documentation?

**Table 1:** Exception preconditions inferred by wit

				expres			maybes	
project	hash	kloc	time	#	m	p	?#	?p
com/sun	–	30	–	55	48	1.0	78	–
sun	–	128	–	566	474	1.0	1 068	–
java	–	209	–	3 420	2 578	1.0	1 666	–
javax	–	8	–	190	145	1.0	41	–
jdk	–	52	–	847	742	1.0	598	–
**overall JDK**	da75f3c4ad5	428	765	5 078	3 987	1.0	3 451	0.36
accumulo	7db0561cac	33	311	995	908	1.0	1 335	0.3
Activiti	31024bc756	103	150	685	543	1.0	212	0.2
asm	72e8ec49	28	130	203	126	1.0	428	0.8
asterisk-java	5c56735c	30	27	27	24	1.0	46	0.4
AutomatedCar	c137e56a	4	2	2	2	1.0	4	0.5
Baragon	10660b41	15	6	10	10	1.0	50	0.2
bigtop	ee28ba88	6.5	4	9	9	1.0	6	0.2
byte-buddy	4c57c80aab	57	974	356	348	1.0	374	0.8
camel	0a735ae926c	972	2 626	1 558	1 276	1.0	1 111	0.4
closure-compiler	fe0cebacd	287	538	158	157	1.0	654	0.2
commons-bcel	f1a1459f	35	137	76	74	1.0	896	0.4
commons-configuration	1b406c17	20	12	170	139	1.0	53	0.4
commons-io	2ae025fe	9.5	23	240	187	1.0	186	0
commons-lang	90e0a9bb2	29	55	611	484	1.0	230	0.8
commons-math	674805c64	61	264	1 078	612	1.0	573	0.8
commons-text	21fc34f	10	32	235	156	1.0	138	0.6
Confucius	e375cb9	0.5	1	45	18	1.0	14	0.4
curator	9aafdec9	26	35	192	116	1.0	126	0.6
dubbo	b5e65a6d2	99	274	413	341	1.0	225	0.4
flink	db248b2176	568	1 245	5 661	4 059	1.0	5 201	0.8
gae-java-mini-profiler	9cb1ba6	0.5	1	0	0	–	0	–
h2database	0ee51f54a	150	229	526	507	1.0	834	0.6
httpcomponents-client	29ba623eb	32	37	27	24	1.0	90	0.4
itext7	ae78654a5	145	880	681	522	1.0	702	0.7
jackrabbit	35d5732bc	260	300	1 224	1 111	1.0	1 595	0.8
jackrabbit-oak	f8c7b551a4	26	334	502	493	1.0	667	0.4
jackson-databind	972d5a28a	63	57	180	166	1.0	153	0.6
jfreechart	5aac9ae4	84	133	1 387	1 149	1.0	800	1.0
jmonkeyengine	499e73ab0	19	376	634	569	1.0	1 220	0.2
joda-time	27edfffa	29	58	250	228	1.0	355	0.6
logging-log4j2	59f6848b7	99	159	472	304	1.0	392	0.2
lucene-solr	7ada4032180	685	1 545	3 380	2 755	1.0	4 132	0.6
pdfbox	01bce4dde	106	230	255	239	1.0	362	0.2
poi	270107d9e	260	403	710	624	1.0	1 851	0.2
santuario-xml-security-java	86179876	35	38	167	142	1.0	131	0.6
shiro	0c0d9da2	27	39	154	141	1.0	145	0.2
spoon	34c23fc7	75	86	272	268	1.0	357	0.4
spring-cloud-gcp	6c95a16f	20	20	13	13	1.0	10	0.8
spring-data-commons	4acd3b70	28	24	31	29	1.0	123	0.4
swingx	9e33bc0	72	108	157	149	1.0	217	0.8
traccar	eac5f4889	54	60	2	2	1.0	76	0
visualee	88732d9	1.8	3	0	0	–	3	0
weiboclient4j	80556b1	7.8	10	6	6	1.0	9	0.2
wicket	7c0009c8df	109	1 069	930	811	1.0	656	0.6
wildfly-elytron	3457737d98	80	128	340	316	1.0	233	0.2
xmlgraphics-fop	7edce5dd5	165	940	385	318	1.0	617	0.6
**overall other projects**	–	5 720	14 116	25 409	20 474	1.0	27 592	0.5
**overall**	–	6 148	14 881	30 487	24 461	1.0	31 043	0.5

For each analyzed project: the short git commit hash; the size of the analyzed source code in thousands of lines (kloc); wit’s total running time in minutes; the number # of inferred exception preconditions (expres), the number m of methods and constructors with some inferred exception preconditions, the precision p based on a manual analysis of a sample, the number ?# of maybes exception preconditions, and the percentage ?p of these that are correct based on a manual analysis of a sample

### Experimental Subjects

In our evaluation, we ran wit on two groups of projects: several standard libraries in Java’s JDK and 46 open-source Java projects surveyed by recent papers investigating the (mis)use of Java library APIs Wen et al. (2019); Zhong et al. (2020); Kechagia et al. (2021) and the automatic generation of tests for some of these libraries Nassif et al. (2021). Table 1 lists all our experimental subjects.

**JDK modules** The JDK (Java Development Kit) includes arguably Java’s most widely used and mature libraries, featuring virtually in every Java project Nassif et al. (2021); Kechagia et al. (2019) and abundantly documented. We selected JDK 11^24^ to run our experiments, since it’s the most recent LTS (Long Term Support) release that JavaParser can handle at the time of writing. Given the JDK’s gargantuan size and complexity, we selected five of its modules (subdirectories of java.base/share/classes) and ran wit on all of them as if it were a regular Java project: modules com/sun, java, javax, sun, and jdk.

**Other projects** The other group of 46 experimental subjects includes several projects that are also large, widely-used, mature Java projects in various domains (base libraries, GUI programming, security, databases)—especially the 26 projects from the Apache Software Foundation, which recent empirical research has shown to be extensively documented and thoroughly tested Zhong et al. (2020); Nassif et al. (2021). On the other hand, a few projects taken from Kechagia et al. (2021) are smaller, less used, or both. For instance, projects gae-java-mini-profiler, visualee, and AutomatedCar are no longer maintained. This minority of projects makes the selection more diverse, so that we will be able to evaluate wit’s capabilities in different scenarios.

We used the latest commit/stable release in every project, at the time of writing, with two exceptions: Apache lucene-solr was recently split into two separate projects, and thus we used the last version before the split; we analyzed version 2.6 of Apache Commons IO to match Nassif et al. (2021)’s thorough manual analysis—which we used as ground truth to answer RQ2.

### Experimental Setup

We ran wit on the source code of all projects, after excluding directories that usually contain tests (e.g., +src/test/+) or other auxiliary code. All experiments ran on a Windows 11 Intel i9 laptop with 32GB of RAM. By default, wit only infers the exception preconditions of *public* methods; if a public method calls a non-public one, wit will also analyze the latter, but will report only public exception preconditions. wit analyzes each class in isolation; then, it combines the results for all classes in the same project and outputs them to the user.

Unless we explicitly state otherwise, wit ran with default options in the experiments. In particular, it performed *modular analysis* (described in Section 3.4); therefore, we first ran wit on the JDK modules, then on the Apache Commons libraries (lang, io, text, math, configuration, in this order) followed by all other projects in alphabetical order. Since practically all projects use some JDK libraries, and several projects also use Apache Commons libraries, this execution order maximizes the chances that wit can reuse the results of one of its previous runs to perform an effective modular analysis. In contrast, client-of dependencies between projects other than the JDK and Apache Commons libraries are more sparse; therefore, the alphabetical order is somewhat arbitrary, but even following a different order is unlikely to significantly affect wit’s capabilities.

To answer **RQ1 (precision)**, we performed a manual analysis of a sample of all exception preconditions reported by wit to determine if they correctly reflect the exceptional behavior of the implementation. The first author tried to map each inferred exception precondition to the source code of the analyzed method. In nearly all cases, the check was quick and its outcome clear. The few exception preconditions whose correctness was not obvious were analyzed by the other author as well, and the final decision was reached by consensus. We were conservative in checking correctness: we only classified an exception precondition as correct if the evidence was clear and easy to assess.

To answer **RQ2 (recall)**, we used Nassif et al. (2021)’s dataset—henceforth, DSc—as ground truth. DSc includes 844 manually-collected exception preconditions^25^ (expressed in structured natural language, e.g. “if offset is negative”) for all public methods in Apache Commons IO’s base package collected from all origins (package code, libraries, tests, documentation, ...). We counted the exception preconditions inferred by wit that are semantically equivalent to any in DSc. Matching DSc’s natural-language preconditions to wit’s was generally straightforward, as we didn’t have to deal with subtle semantic ambiguities: since wit only reports correct exception preconditions as expres, we only had to match (usually simple) natural-language expressions to their Java Boolean expression counterparts.

Using DSc as ground truth assesses wit’s recall in a somewhat restricted context: (i) DSc targets exclusively the Commons IO project, whose extensive usage of I/O operations complicates (any) static analysis; (ii) DSc describes all sorts of exceptional behavior, including the “not typically documented” runtime exceptions Nassif et al. (2021). To assess wit’s recall on a more varied collection of projects, we also considered Zhong et al. (2020)’s dataset—henceforth, DPA—which includes 503 so-called “parameter rules” of public methods in 9 projects (a subset of our 46 projects described in Section 4.1). A parameter rule is a pair $\langle m, p \rangle$, where *m* is a fully-qualified method name and *p* is one of *m*’s arguments; it denotes that calling *m* with some values of *p* may throw an exception. Important, parameter rules do not express the *values* of *p* that determine an exception, and hence they are much less expressive than preconditions; however, they are still useful to determine “how much” exceptional behavior wit captures. We counted the exception preconditions inferred by wit that match DPa: a precondition *c* matches a parameter rule $\langle m, p \rangle$ if *c* is an exception precondition of method *m* that depends on the value of *p*. This is a much weaker correspondence than for DSc, but it’s all the information we can extract from DPa’s parameter rules.

To better characterize the exception preconditions that wit could *not* infer, we performed an additional manual analysis of: (a) 746 of DSc’s exception preconditions among those that wit did not infer and (a) 218 exception preconditions reported by wit as “maybe” (that is, which may be incorrect). These 964 additional cases help assess what it would take to improve wit’s recall.

To answer **RQ3 (features)**, during the manual analysis of precision we also classified the basic features of each exception precondition *r* of a method *m*. We determine whether *r* corresponds to an exception that is thrown directly by *m* or propagated by *m* (and thrown by a called method). We count the number of Boolean connectives 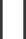 and 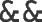 in *e*, which gives an idea of *r*’s complexity. Then, we determine if each subexpression *e* of *r* constraints *m*’s *arguments*, or *m*’s object *state*; and we classify *r*’s check according to whether it is: (a) *null* check (whether a value is null), (b) a *value* check (whether a value is in a certain set of values), (c) a *query* check (whether a function call returns certain values). For example, here are expressions of each kind for a method m with arguments int x and String y, whose class includes fields int[] a, int count, and method boolean active():

**Table:** 

void m(int x, int[] y)	*argument*	*state*
*null*	y == null	this.a != null
*value*	x == 1	this.count> 0
*query*	y.isEmpty()	!this.active()

An exception precondition may combine expressions of different kinds; for instance, 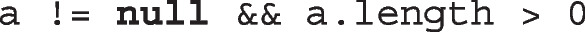 combines a null and a value check.

To answer **RQ4 (modularity)**, we ran wit again on 5 projects with modular analysis *disabled*, and compared wit’s output on these projects with and without modular analysis. We selected the 5 projects from diverse domains, which demonstrate using different JDK libraries and methods. Besides comparing the number of reported exception preconditions with and without modular analysis, we manually inspected 75 maybes: (a) For each project, among methods for which both the modular and non-modular analysis reported *some* maybes, we randomly picked 6 maybes reported by the non-modular analysis and 6 maybes reported by the modular analysis for the same methods;^26^ this sample of 60 maybes ($6 \times 5 \times 2$) gives us an idea of how maybes change when modular analysis is enabled. (b) For each project, among methods for which *only* the modular analysis reported some maybes, we randomly picked 3 maybes; this sample of 15 maybes ($3 \times 5$) demonstrates cases where the modular analysis strictly outperforms the non-modular one.

To answer **RQ6 (usefulness)**, we first inspected the source code documentation (Javadoc and comments) of all methods with exception preconditions analyzed to answer RQ1, looking for mentions of the thrown exception types and of the conditions under which they are thrown.

We focused on Javadoc documentation: while we also considered non-structured comments a priori, all cases of documented exceptional behavior that we found used at least some Javadoc syntax.

We also selected 90 inferred exception preconditions among those that were not already documented, and submitted them as 8 pull requests in 5 projects: Accumulo,^27^Commons Lang,^28^^,^^29^^,^^30^Commons Math,^31^^,^^32^Commons Text,^33^ and Commons IO.^34^ We selected these five projects as they are very active and routinely spend effort in maintaining a good-quality documentation. Each pull request combines the exception preconditions of methods in the same class or package, and expresses wit’s exception preconditions using Javadoc @throws tags. To compile each pull request, we sometimes complemented the Javadoc with a brief complementary natural-language description, and possibly some tests (expressing wit’s example inputs in the form of unit tests). We also tried to adjust the Javadoc syntax to be consistent with each project’s style (for example, expressing a != null as either a not null or @code a != null). In all cases, reformulating wit’s output was a trivial matter.

## Experimental Results

As described in Section 3.6, wit produces two kinds of exception preconditions. The main output are those whose feasibility was fully checked (Section 3.5); others are marked as *maybe* and can still be correct but have no guarantee. As done in previous sections, we call “expres” the former and “maybes” the latter. Unless explicitly stated otherwise, the term “project” denotes any of the 51 experimental subjects (Section 4.1): one of the 5 JDK modules or one of the 46 open-source projects we analyzed.

### RQ1: Precision

Overall, wit reported 30 487 expres and 31 043 maybes in 40 263 methods (24 461 methods with some expres and 17 564 with some maybes)—out of a total of 460 032 analyzed public methods from 59 733 classes in 51 projects.

In order to validate wit’s feasibility check, we manually analyzed a sample of 742 expres to determine if they are indeed correct. This sample size is sufficient to estimate precision with up to 5% error and 99% probability with the most conservative (i.e., 50%) a priori assumption authorname (1999); thus, it gives our estimate good confidence without requiring an exhaustive manual analysis Zhou et al. (2020); Nassif et al. (2021). We applied stratified sampling to pick the 742 expres: we randomly sampled 10 instances in each of the 49 projects where wit detected some expres.^35^ This manual analysis found that *all* expres were indeed correct, that is 100% precision.

As we explained in Section 3, wit’s maybes still have a chance of being correct exception preconditions, but they remain educated guesses in general. We randomly picked 218 maybes uniformly in the 50 projects that report some^36^

and manually checked them as we did for the expres. We found that 47% (102) of them are indeed correct; thus, wit’s precision remains high (88% $= (102+742)/(218+742)$) even if we consider all maybes. As we further discuss in Section 5.2, in most cases, wit could not confirm the maybes as correct because they involve unsupported Java features (see Section 3.7). 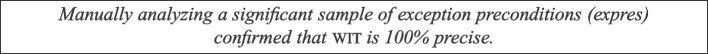

**Table 2:** wit’s recall using two datasets DSc and DPa (described in Section 4.2) as ground truth. For each project, # is the dataset’s total number of exception preconditions (DSc) or parameter rules (DPa); the other columns reports the percentage correctly inferred by wit: E only considers expres, E+M expres and maybes; all considers all exception items; supported only those with features wit supports

			all		supported	
dataset	project	#	E%	E+M%	E%	E+M%
DSc Nassif et al. (2021)	commons-io	844	9	12	57	72
DPa Zhong et al. (2020)	asm	54	6	23	25	75
	commons-io	65	77	78	94	96
	jfreechart	42	80	85	84	89
	**overall**	1,345	13	23	48	84

### RQ2: Recall

We compute the recall on both datasets DSc and DPa in four ways: considering only expres or also maybes; and considering only wit’s supported features or all Java features. Table 2 summarizes the results that we detail in the following.

#### Dataset DSc

Out of DSc Nassif et al. (2021)’s 844 manually identified exception preconditions, wit detected 77 expres in 6 classes of Commons IO (1 in FileNameUtils, 4 in LineIterator, 15 in IOUtils, 8 in FileCleaningTracker, 44 in FileUtils, 3 in HexDump, and 2 in ByteOrderMark), that is a recall of 9% (77/844). However, 708 out of DSc’s 844 exception preconditions are of kinds unsupported by wit (see Section 3.7). After excluding unsupported exception precondition kinds,^37^wit’s recall estimate becomes 57% ($77/(844-708)$).
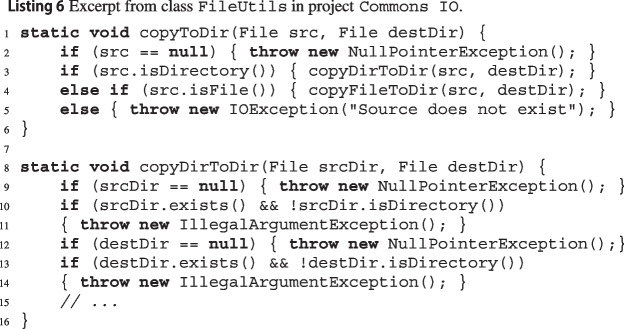


To better understand wit’s recall, we analyzed the 708 Commons IO exception preconditions from DSc that wit didn’t report as expres. We can classify these missed preconditions in two groups.

- *Unsupported features:* As mentioned, the largest group of missed preconditions (547 or 77% of the missed preconditions) involve Java language features that wit does not support.
- *Implicit exceptions:* Another group of missed preconditions (161 or 23% of the missed preconditions) correspond to implicit exceptions that are thrown by the Java runtime (e.g., when a null pointer is dereferenced), which we deliberately ignore (as discussed in Section 3.7). A significant case is class EndianUtils^38^ for which DSc reports 48 exception preconditions involving ArrayIndexOutOfBounds or NullPointer exceptions thrown implicitly.

#### Dataset DPa

Using 175 parameter rules^39^ of DPa Zhong et al. (2020)’s dataset as reference suggests that wit’s recall varies considerably depending on the characteristics of the analyzed project. Overall, wit inferred 85 matching expres and 8 matching maybes, corresponding to a recall of 49% (expres only) and 53% (expres+maybes). If we exclude the parameter rules involving features unsupported by wit, the recall becomes 71% (expres only) and 78% (expres+maybes). wit struggles the most on projects like asm, which extensively uses features and coding patterns^40^ that wit currently doesn’t adequately support: as a result, wit’s recall is fairly low (considering all parameter rules, 6% with expres only and 23% with expres+maybes; considering only supported ones, 25%/75%). In contrast, more “traditional” Java projects like JFreeChart^41^ extensively follow programming practices such as validating a method’s input, which are a better match to wit’s current capabilities: as a result, wit’s recall is quite high (considering all parameter rules, 80% with expres only and 85% with expres+maybes; considering only supported ones, 84%/89%). 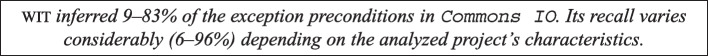


### RQ3: Features

Section 5.2’s comparison of wit’s preconditions with those in DSc Nassif et al. (2021)’s extensive collection confirmed what also reported by other empirical studies Blasi et al. (2018); Zhou et al. (2020): exception preconditions are often concise and structurally simple. This was also reflected in a manual sample of 412 expres inferred by wit,^42^ which we manually inspected to determine their features. In terms of size, 74% of them are simple expressions without Boolean connectives 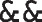/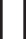; and only 7% include more than one connective. In terms of control-flow complexity, 68% of wit’s expres involve exceptions that are thrown directly by the analyzed method (as opposed to propagated from a call).

Over 70% of all expres constrain a method’s arguments (65% constraint *only* the arguments), whereas about 24% predicate over object state. null checks are more frequent (49% of expres), followed by value checks (40% of expres); and 81% of expres have either or both. In contrast, query checks are considerably less frequent (11% of expres include one).

These features are a combination of the intrinsic characteristics of exception preconditions, and wit’s capability of detecting them.

If we look at maybes, they tend to include query checks more frequently (50%), which is to be expected since a method call can be soundly used in a precondition only when it is provably pure (Section 3.6).

Up to 12% of the expres in the sample are the simplest possible Boolean expression: true. Nine of 13 expres of spring-cloud-gcp are of this kind. These usually correspond to methods that unconditionally throw an UnsupportedOperation exception to signal that they are effectively not available;^43^,^44^

see project lucene-solr’s class ResultSetImpl for an example.^45^ In Java, this is a common idiom to provide “placeholders,” which will be replaced by actual implementations through overriding in subclasses. While this is a common programming pattern that leverages polymorphism, it nominally breaks behavioral substitutability Liskov and Wing (1994); Nguyen et al. (2014): a method’s precondition should only be weakened Meyer (1997), but no Boolean expression is weaker than true.

Some of the exception preconditions that we manually inspected revealed interesting and non-trivial features. wit could infer expres embedded in complex expressions, such as in the case^46^ of an empty string that triggers an exception in the “else” part e of a ternary expression. c ? t : e. It also followed method calls collecting complex conditions and presenting them in a readable, simplified form. For example, for a ConcurrentModification exception,^47^ or after collecting constant values from other classes.^48^

We also found examples of exceptional behavior documented in Javadocs in a way that mirrors wit’s output, such as “IndexOutOufBoundsException $\mathtt {i < 0}$ or $\mathtt {i >}$ array.length”.^49^

In all, wit’s output is often concise and to the point—and thus readable and useful. 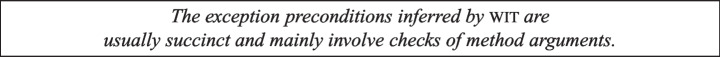


### RQ4: Modularity

To answer RQ4 (the impact of modular analysis), Section 5.4.1 first discusses how the output of wit changes when modular analysis is disabled vs. when it is enabled; then, Section 5.4.2 presents the results of a manual comparison of a sample of exception preconditions obtained with and without modular analysis.

#### Exception Preconditions in Modular vs. Non-Modular

Table 3 presents the results of the comparison between wit running with and without modular analysis (Section 3.4) on five of the projects used in our experimental evaluation.

**Running time** In terms of running time, modular analysis usually leads to an increase of running time (32% longer on average); this is to be expected, since modularity generally increases the number of paths that are analyzed by wit, as it “extends” them with information about methods analyzed in a different run.

**Effectiveness** Modular analysis usually brings a modest (but non-trivial in absolute numbers) increase in the number of expres reported by wit (2% more on average). These cases correspond to exceptional paths that include calls to external methods: in the non-modular analysis, these paths may only lead to maybes; in contrast, in the modular analysis, wit has enough information to completely and correctly reconstruct the exceptional behavior about these paths, thus reporting expres.

Modular analysis usually brings a much bigger increase in the number of maybes (156% more on average): since maybes have no guarantee of correctness, using a maybe in a library to reason about a call within a caller method is quite likely to determine an additional maybe in the caller—which also may or may not be correct.

**When modular analysis is counterproductive** However, modular analysis does not always lead to detecting more expres; for example, wit reported 1–2% *fewer* expres in projects jfreechart and pdfbox when enabling modular analysis. This happens because modular analysis replaces a call to an opaque method with whatever exception path wit extracted from the called method. In some cases, the called method’s exception precondition may be a very partial approximation of the callee’s full exceptional behavior; therefore, using it in place of the call may be counterproductive to obtain a provably feasible exception precondition in the caller. In fact, this is a common problem of modular reasoning Tschannen et al. (2014): if the callee’s specification is weak, there is very little we can conclude about the caller’s behavior.

Our manual analysis indicates that the overwhelming majority of cases where using modular reasoning led to *fewer* expres involved methods calling string methods such as String.length() and String.equals(). For example, when wit analyzes String.equals()’s implementation in the JDK,^50^ it encounters several features and special cases that limit its effectiveness, such as different string encodings^51^ and compacted strings;^52^ furthermore, the Java runtime represents a String as a byte array,^53^ a type that wit does not currently support. As a result, wit only reports some very narrow, overly complex exception paths for String.equals(), corresponding to the few paths within its implementation that do not depend on any of those complex language features. What happens when wit processes a method such as the one in Listing 7, which makes numerous calls to String.equals(), with modular analysis enabled? Replacing the calls with the previously extracted exception paths leads to an overly narrow, needlessly complex path condition, which bogs down the SMT solver and does not lead to any provably feasible path in the caller. In contrast, if modular analysis is disabled, wit simply encodes the calls to String.equals() as Boolean variables with basic constraints, which is sufficient in some cases to get to a working proof of feasibility—and hence to an expre correctly characterizing setHighlightingMode’s^54^ exceptional path.

**Table 3:** Impact of using wit’s modular analysis (Section 3.4) for five projects

		expres		maybes
project	$\Delta$ time	$\Delta$ #	$\Delta$ m	$\Delta$ ?#
camel	1.17	1.04	1.03	3.51
commons-io	0.65	1.03	1.06	1.81
commons-lang	2.30	1.05	1.05	6.21
jfreechart	1.70	0.99	1.01	0.91
pdfox	2.67	0.98	0.97	2.71
**overall**	1.32	1.02	1.02	2.56

For each project, we consider the same measures as Table 1: the overall running time, the number # of reported expres, the number m of methods for which wit reported at least one expre, and the number ?# of reported maybes. Each column $\Delta X$ reports the ratio between *X* measured with modular analysis and *X* measured without modular analysis; for example, wit reports 4% more expres (1.04) in project camel when modular analysis is enabled


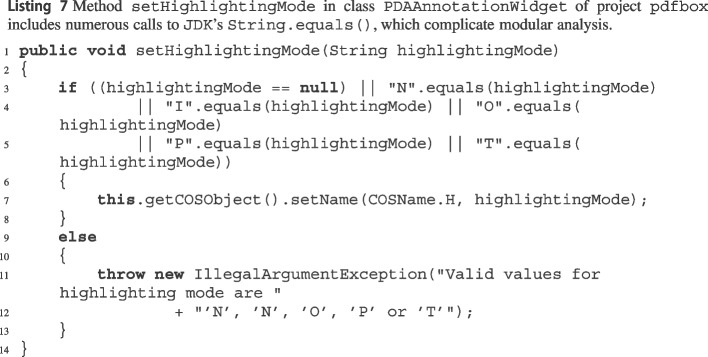


#### Correctness of Maybes in Modular vs. Non-Modular

We first sampled 30 methods where both the non-modular and modular analysis reported *some* maybes, and inspected one maybe in each case (for a total of $30 + 30 = 60$ maybes). In the non-modular analysis, 27 (90%) of the 30 maybes were correct; in the modular analysis, 16 (53%) of the 30 maybes were correct. Then, we sampled 15 other methods where *only* the modular analysis reported *some* maybes, and inspected one maybe in each case (for a total of 15 maybes). Only 4 (27%) of the 15 maybes were correct.

These results suggest that wit’s modular analysis is usually less reliable at inferring (correct) maybes. This is in contrast to the inference of expres, which are correct by construction. In all, unless one wants to maximize the output of reported maybes, it may be preferable to only perform modular analysis for expres, excluding maybes.

This inferior performance of the non-modular analysis is usually due to complex language features used in the JDK or other called libraries that wit does not adequately support; in these cases, the non-modular analysis’s approach of treating these calls as black boxes is more likely to avoid generating incorrect maybes than the modular approach that reuses probably inconsistent or mismatched maybes extracted when analyzing the called libraries.

Let us discuss a few concrete examples of language features that led to incorrect maybes with the modular analysis. One is the complex behavior of floating-point arithmetic (type Double in Java); wit’s simple encoding of numbers cannot deal with special values such as NaN^55^ and Inf (obtained, for example, when dividing 1.0 by 0.0^56^). Another one is the JDK’s Collections Framework, which would require a suitable (non-trivial) logic encoding in Z3 to work in wit.

A different kind of problem occurred when analyzing data-structure methods such as the JDK’s Stack.pop,^57^ which throws an exception when the stack is empty. wit reports a correct exception precondition for pop; however, the precondition expression mentions a protected field^58^ used in Stack’s internal representation.^59^ As a result, the exception precondition is not usable correctly to analyze clients of the Stack class, such as in one of the maybes we inspected for project pdfbox.^60^ To handle such cases Zeng et al. (2021), one could try to convert any references to private members into calls to public getter methods—if they are available.

It remains that wit’s modular analysis increases the number of expres in most projects. We found a few cases where some exception preconditions reported as maybe by the non-modular analysis became an expre in the modular analysis. One such cases was class IntersectionResult’s constructor^61^ in project Commons Text. As you can see in Listing 8, the exception path that ends at line 10 involves a call to the JDK’s Math.min function. Without modular analysis, wit can only report the whole conditional expression 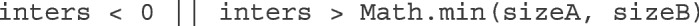 as a maybe. In contrast, wit’s modular analysis can recover Math.min’s behavior from its previous analysis of the JDK; thus, it reports two correct expres for the same exceptional path:

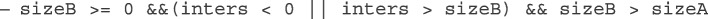
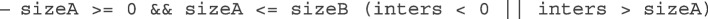

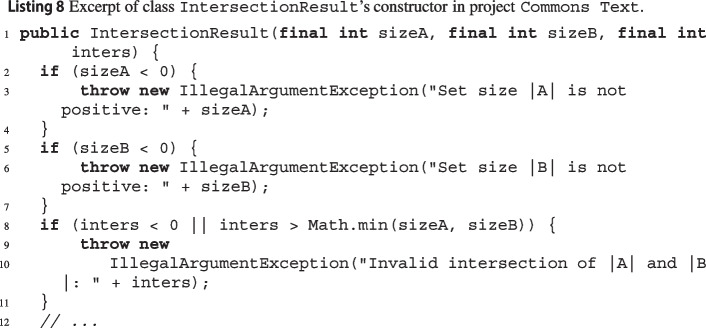

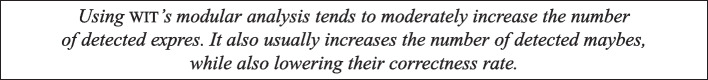


### RQ5: Efficiency

Thanks to the heuristics it employs (Section 3.7) and to the nature of exception preconditions wit can infer (which tend to be simpler compared to general program behavior), wit’s analysis is quite lightweight and scalable. As shown in Table 1, its running times are generally short: it processed the entire Apache Commons Lang in just 55 minutes—17 seconds on average for each of the project’s 200 top-level classes. It also scales well to very large projects: it analyzed the 9 780 classes of Apache Camel (the largest project in our collection) in 44 hours—just 16 seconds per class on average. Key to this performance is wit’s capability of analyzing each class in isolation, without requiring any compilation or build of the whole project.

Take method ASMifier.appendAccess()^62^ as an example of how wit’s heuristics are useful. It is from project ASM and embedded under the internal subdirectory of the JDK. The method has several nested if-else branches, that lead to millions of paths. wit’s heuristics are crucial to avoid getting bogged down analyzing such complex pieces of code. 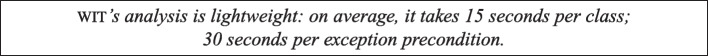


### RQ6: Usefulness

This section discusses to what extent wit’s exception preconditions and the documented exceptional behavior of methods overlap. We first look into all projects except the JDK modules (Section 5.6.1), and then analyze the JDK separately (Section 5.6.2); finally, we discuss how we submitted some of wit’s inferred exception precondition as pull requests (Section 5.6.3).

#### Usefulness: Regular Projects

Let us first focus on the 46 projects in Table 1 excluding the JDK modules. We analyzed a subset sample of 517 expres and maybes that wit correctly inferred for these projects; 72% (374) of them are not documented; precisely, 242 of them belong to methods without any Javadoc, and 120 to methods with some Javadoc that does not describe that exceptional behavior. In contrast, 27% (138) of wit’s exception preconditions are properly documented; and 6% (29) of them are only partially documented (usually with a @throws Exception tag that does not specify the conditions under which an Exception is thrown).

wit’s inferred preconditions can substantially improve even the cases of partial documentation. An example is Apache Curator’s method validatePath(String),^63^ whose Javadoc just says that “@throws IllegalArgumentException if the path is invalid”. wit detects several different exception preconditions for when an exception of class IllegalArgumentException is thrown: a path is invalid when it is null, empty, not starting or ending with a /, etc.

Scenarios (such as the one in Section 2.2) where a method propagates an exception thrown by one of its callees may be hard to characterize precisely (especially when the callees’ exceptional behavior is not documented); wit’s analysis can be particularly valuable in these cases. Indeed, 36% (187) of wit’s 517 exception preconditions analyzed in this section involve *nested* exception preconditions; only 24% (47) of these 196 exception preconditions are documented. This corroborates Raymond and Weimer (2008)’s finding that Javadocs rarely mention exceptions thrown by called methods.

Section 5.2’s manual analysis of recall further surfaced evidence of wit’s practical usefulness. Even though the DSc dataset (which we used as ground truth to assess recall) is a paragon of comprehensiveness, wit’s modular analysis still managed to detect exception preconditions that were missed by DSc’s painstaking manual analysis. Listing 9 shows Commons IO’s method FileUtils.copyURLToFile(),^64^ which calls methods from JDK class URLConnection^65^^,^^66^. Commons IO’s documentation of this method mentions five conditions under which the method will throw an IOException. The DSc dataset reports another two exception preconditions that trigger implicitly a NullPointerException. However, only wit found that that the calls to setConnectTimeout and to setReadTimeout will throw an IllegalArgumentException if their argument is a negative integer. This is yet another example that manually detecting and documenting exception preconditions is tedious, time-consuming, and error prone; thus, the kind of automation provided by wit can be very useful.
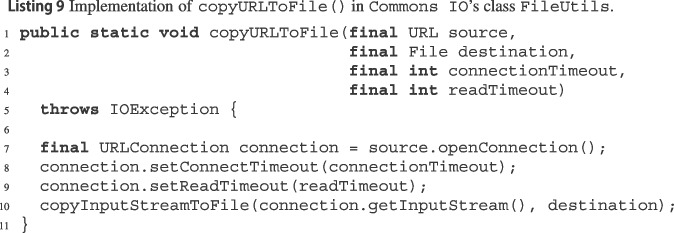


#### Usefulness: JDK Modules

We analyze the JDK separately, since it is arguably Java’s most thoroughly documented library Zhou et al. (2020); Kechagia et al. (2019); therefore, it is natural to expect that a higher fraction of wit’s inferred exception preconditions will also feature in the JDK’s official Javadoc documentation.

We analyzed a subset sample of 361 expres and maybes that wit correctly inferred for the JDK; 38% (136) are not documented. We also found that 48% (172) of the 358 preconditions occur in nested calls (when an exception is propagated from a method call); and 61% (106) of them are documented, which is significantly higher than the ratio for the other projects.

Even though the JDK’s documentation is generally outstanding, we found inconsistencies in when and how it documents exceptional behavior. For example, it sometimes only documents a subset of all possible unchecked exceptions a method may throw;^67^ or occasionally uses the throws keyword to declare (unchecked) runtime exceptions.^68^$$^{,}$$^69^ JDK’s package Time^70^ uses a distinctly different style of documenting NullPointerExceptions, which betrays the package’s origins as a derivative of project joda-time; to declare that a method thows a null pointer exception when one of its parameters p is null, it writes:@param p $$\mathtt {<}$$description of p$$\mathtt {>}$$, not null.^71^ Incidentally, project JFreechart uses a similar style of documentation.

Another interesting finding in the JDK is that older modules are more likely to neglect using exception *messages*—which, however, can provide valuable debugging information Marcilio and Furia (2021). For instance, classes introduced in versions 1.0^72^ and 1.1^73^ always instantiate NullPointerException without arguments (i.e., no message). Despite these outliers, the JDK generally tries to use expressive exception messages, and to improve their clarity. For example, Integer.parseInt throws a null pointer exception with an uninformative message "null" in JDK 11;^74^ in JDK 17, however, the maintainers changed it to the more informative "Cannot parse null string".^75^
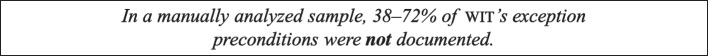


#### Improving Project Documentation Using wit

While there may be situations where documenting every source code method is not needed or recommended, properly documenting *public* methods of APIs (remember that all of wit’s exception preconditions refer to public methods) is an accepted best practice Zhou et al. (2020); Nassif et al. (2021).

Indeed, there is evidence that several of the projects used in our evaluation (Section 4.1) routinely improve their Javadoc documentation of exceptions,^76^^,^^77^ and often recommend^78^ or even require^79^^,^^80^ accurate Javadocs in any code contributions.

To determine whether wit’s inferred preconditions can be a valuable source of API documentation, we collected 90 exception preconditions extracted by wit in 5 Apache projects and submitted them as 8 pull requests (as described in Section 4.2). At the time of writing, maintainers accepted (without modifications) 6 pull requests containing 81 preconditions—63 (78%) of them occurring in nested calls. Two pull requests to project Commons Math have not been reviewed yet. Interestingly, one to project Commons Lang was on hold for several months because the project maintainers realized that the 10 methods whose exceptional behavior we document are inconsistent in using IllegalArgumentException vs. NullPointerException, and they preferred to fix this inconsistency before updating the documentation.

When submitting our improvements to project Commons Lang, we opened a JIRA issue^81^ sharing our findings. Several months after our initial pull request, a GitHub user submitted four Javadoc modifications in a new pull request^82^ that mentioned our JIRA issue. Shortly afterwards, a Commons Lang maintainer asked us to review the modifications in the new pull request, and suggested that we submit all our findings (i.e., all the exception preconditions that could be included in the documentation) in order to close the JIRA issue. In the end, we worked together with the author of the latest pull request to submit 89 wit exception preconditions (27 new pieces of Javadoc documentation and 62 fixing existing documentation), as well as tests for 9 classes. All of the exceptions from the additions and fixes occur in nested calls, which may explain why they went undetected for a long time. The pull request was accepted in the same day and merged ten days later.

Overall, our 9 pull requests (8 initial ones, plus the latest one suggested by the maintainers) include 189 exception preconditions (90 in the initial batch, and 89 in the latest one). These pull requests contain 157 (88%) preconditions occurring in nested calls; 61 (34%) that refer to missing documentation, and 118 (66%) that target a wrongly documented exception. A total of 170 preconditions (81 in the initial batch, and 89 in the latest one—or 95% of all those submitted) were merged into the projects’ official documentation.

It is significant that the projects that accepted these pull requests are known for their extensive and thorough documentation practices Zhong et al. (2020); Nassif et al. (2021). The fact that wit could automatically detect several exception preconditions that were missing from their documentation, and promptly added following our pull requests,^83^ indicates that wit’s output can be quite useful. We expect that wit’s precise output can have an even bigger impact on scarcely documented projects. 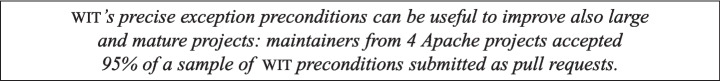


## Threats to Validity

The main threat to the *internal validity* of our assessment of wit’s *precision* (Section 5.1) comes from the fact that it is based on manual inspection of Java code and documentation. Like all manual analyses, we cannot guarantee that no mistakes were made. Nevertheless, various evidence corroborates the claim that wit’s precision is high. First, wit’s precision follows from its design; therefore, the manual analysis was primarily a validation of wit’s *implementation*, checking that no unexpected source of incorrectness occurred in practice. Second, we inspected not only the source code but also any official documentation, tests, as well as the datasets of related studies of Java exceptions Marcilio and Furia (2021); Nassif et al. (2021). Third, the authors extensively discussed together the few non-obvious cases, and were as conservative as possible in the assessment. We followed similar precautions to mitigate threats to our assessment of wit’s *recall* (Section 5.2), where we relied on Nassif et al. (2021)’s and Zhong et al. (2020)’s manual analyses as ground truth.

As customary Zhou et al. (2020), we assume that the implementations of all analyzed methods are correct: wit’s goal is to capture an implementation’s exceptional behavior as faithfully as possible; detecting bugs in such implementations is out of its (current) scope.

Our selection of 46 Java projects includes several very popular Java open source libraries, which were used in recent related work, and in addition several modules in Java’s official JDK; this helps reduce threats to *external validity*. It remains that the exceptional behavior of libraries may be different than that of other kinds of projects. Since library APIs tend to perform more input validity checks Robillard et al. (2013), it is possible that wit would report fewer exception preconditions simply because fewer are present in other kinds of software. Indeed, a handful of the projects with the smallest number of reported expres turned out not to be libraries (see Table 1).

As one of the ground truths to estimate recall, we used a recent survey Nassif et al. (2021) that extensively manually analyzed a single project (Commons IO). As we discuss in Section 5.2, the nature of this project makes it especially challenging for wit, which implies that its recall may be higher on other projects (as the experiments using the other dataset DPa Zhong et al. (2020) suggest).

wit’s implementation has a number of limitations; some reflect deliberate trade-offs, while others could simply be removed by extending its implementation. In its current state, wit has demonstrated to produce useful output and to be precise and scalable.

## Related Work

We first discuss general related work in assertion inference; and then zoom in on a few recent papers that deal specifically with exceptional behavior of Java methods.

**Assertion inference** Automatically inferring preconditions and other specification elements from implementations is a long-standing problem in computer science, which has been tackled with a variety of different approaches. Historically, the first approaches used *static analysis* and thus were typically sound (the inferred specification is guaranteed to be correct, that is 100% precision) but incomplete (not all specifications can be inferred, that is low recall), and may be not applicable to all features of a realistic programming language Cousot and Cousot (1977); Cousot and Halbwachs (1978); Logozzo (2004); Cousot et al. (2013); Seghir and Schrammel (2014). For example, inferring specifications in the form of numeric ranges of values Cousot and Halbwachs (1978) or linear relations between variables Logozzo (2004) is a widespread application of abstract interpretation Cousot and Cousot (1977). Daikon Ernst et al. (2001) was the first, widely successful approach that used *dynamic analysis*, which offers a different trade-off: it is unsound (the “inferred” specifications are only “likely” to be correct) but it is applicable to any program that can be executed. Daikon approach’s practicality also yielded a lot of follow-up work aimed at improving its precision and its flexibility Csallner and Smaragdakis (2006); authorname (2008); Wei et al. (2011), or at combining it with static techniques Csallner and Smaragdakis (2006); authorname (2008); Wei et al. (2011); Csallner et al. (2008); Tillmann et al. (2006); Nguyen et al. (2014). wit is fundamentally based on static analysis, which can be very precise but incomplete Le Goues and Weimer (2009); its heuristics further make it lightweight, and hence applicable to real-world Java projects.

More recently, approaches based on natural language processing (NLP) have gained traction Blasi et al. (2018); Tan et al. (2012); Pandita et al. (2012); Zhong and Su (2013); Wang et al. (2019)—in no small part thanks to the major progress of machine learning techniques on which they are often based. A clear advantage of NLP is that it can analyze artifacts other than program code (e.g., comments and other documentation); on the other hand, machine learning is usually based on statistical models, and hence it cannot guarantee correctness and may be subject to overfitting Phan et al. (2017); Hu et al. (2018).

The work on Toradocu Goffi et al. (2016) and its later extension Jdoctor Blasi et al. (2018) is a relevant representative of the capabilities of natural language processing techniques to extract (exception) preconditions of Java methods. Toradocu/Jdoctor’s preconditions are Java Boolean expressions; thus, they can be directly used to generate test oracles or other kinds of executable specification. In its experimental evaluation on widely used Java libraries, Jdoctor achieved a recall of 83% and a precision of 92%. These high-level results highlight how wit’s and Toradocu/Jdoctor’s approaches are complementary: (a) wit analyzes source code and uses static analysis, which prioritizes accuracy (all expres are correct) at the expense of a lower recall; (b) Toradocu/Jdoctor analyzes Javadoc comments written in (structured) natural language, which cannot provide absolute correctness guarantees, but is often practically effective and achieves a good recall. Another complementary aspect follows from Section 5.6’s observation that only a fraction of the exception preconditions reported by wit are already properly documented—and hence can be automatically extracted with tools like Jdoctor.

Like the “classic” work on static assertion inference, wit extracts preconditions by directly analyzing the behavior of a method’s implementation. An alternative, complementary approach is extracting assertions indirectly by analyzing the *clients* of a method Nguyen et al. (2014); Ramanathan et al. (2007); Wasylkowski and Zeller (2009); Ramanathan et al. (2007); Thummalapenta and Xie (2009); Zhang et al. (2012); Shoham et al. (2007): the patterns used by many clients of the same API are likely to indicate suitable ways of using that API’s methods Robillard et al. (2013).

**Exception preconditions** Buse and Weimer’s work Raymond and Weimer (2008)— which is a refinement of Jex Robillard and Murphy (2003)— shares several high-level similarities with wit: it specifically targets the documentation of exceptional behavior, uses static analysis, and can often improve or complement human-written documentation. Nevertheless, ours and their approach differ in several important characteristics: (a) their approach works on instrumented bytecode, which requires a full compilation of a project to be analyzed (wit only needs the source code of the class to be analyzed); (b) they do not exhaustively check path satisfiability or that only pure method expressions are used in expressions, and hence they may report exception preconditions that are not valid; (c) their evaluation is solely based on a qualitative comparison with human-written documentation, whereas wit’s evaluation quantitatively estimates precision and recall.

SnuggleBug Chandra et al. (2009) is a technique to infer weakest preconditions that characterize the reachability of a goal state from an entry location. Like wit, SnuggleBug is sound and scales to real-world Java projects (even though it works on bytecode and hence requires full project compilation). SnuggleBug’s analysis is more general than wit’s, as it is not limited to *exception* preconditions, and handles calls (including recursion) by synthesizing over-approximated procedure summaries instead of inlining. This approach achieves a different trade-off than wit, which more aggressively gives up on long paths or complex, unsupported language features. SnuggleBug’s evaluation demonstrates one of its main usage scenarios: validating implicit exception warnings.

PreInfer Astorga et al. (2018) infers preconditions of C# programs using symbolic execution (through the Pex white-box test-case generator) by summarizing a set of failing tests’ paths. Compared to wit, PreInfer explores a different part of the assertion inference design space: where wit aims to infer simple preconditions with high precision and scalability, PreInfer focuses on complex preconditions that involve disjunctive and quantified formulas over arrays. These differences in aim are also reflected by the different experimental evaluations: we applied wit to 460 032 methods in 59 733 classes over 46 projects of diverse characteristics and five JDK modules, where it inferred 30 487 preconditions (expres); PreInfer’s evaluation targets 1 143 methods in 147 classes over 4 projects mainly consisting of algorithm and data structure implementations, where it inferred 178 preconditions. Since it relies on Pex, PreInfer’s inferred predicates are only “likely perfect because Pex may not explore all execution paths” Astorga et al. (2018).

A direct, quantitative comparison with these approaches Raymond and Weimer (2008); Chandra et al. (2009); Astorga et al. (2018) is not possible, since their implementations or experimental artifacts are not publicly available.

**Exceptional behavior documentation** Other recent work uses static analysis to extract API specification with a focus on extending and completing programmer-written documentation. PaRu Zhong et al. (2020) is an automated technique that analyzes source code and Javadoc documentation to link method parameters to exceptional behavior. PaRu’s goal is to “identify as many parameter rules as possible [...] it does not comprehend or interpret any rule” Zhong et al. (2020); hence, unlike wit, PaRu does not infer preconditions but just a mapping between parameters and the throw statements that depend on them. PaRu’s empirical evaluation matches this mapping to the available documentation to assess its completeness; it found that 86% of the parameters linked to exceptional behavior are not documented in Javadoc.

Drone Zhou et al. (2020) compares the exceptional behavior of source code to that described in Javadoc in order to find inconsistencies. Similarly to wit, Drone analyzes a program’s control flow statically and uses constraint solving (i.e., Z3)—but to find inconsistencies rather than to analyze feasibility. wit and Drone also differ in some of the Java features they support; for example, Drone keeps track of try/catch blocks (wit misses some paths) but does not follow calls inside conditionals (wit supports them). The several differences between wit’s and Drone’s capabilities reflect their different goals (and, correspondingly, the different research questions of their respective evaluations): Drone aims at finding inconsistencies in whole projects, whereas wit infers preconditions with high precision and nimbly on individual classes. As a result, Drone is run on projects with *some* existing documentation to improve and extend it: the tool “takes API code and document directives as inputs, and outputs repair recommendations for directive defects” (Zhou et al., 2020, §3); wit can run on projects without documentation and reliably find exception preconditions (Section 5.6 showed that many of the manually analyzed exception preconditions found by wit are undocumented).
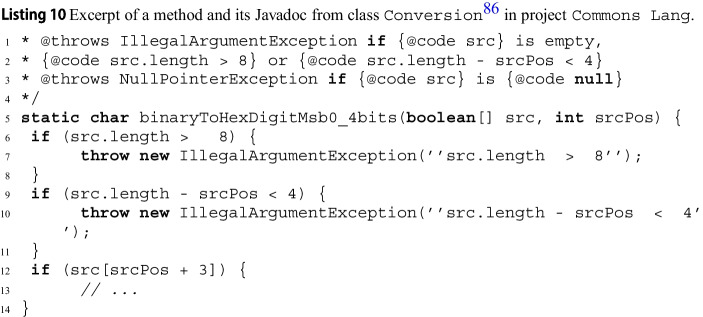


DScribe Nassif et al. (2021) generates unit tests and documentation from manually written templates, which helps keep them consistent. An extensive manual analysis of the exceptional behavior of Apache Commons IO-which we used as ground truth in Section 5.2’s experiments-found that 85% of exception-throwing methods are not documented, not tested, or both, which motivated their template-based approach. wit’s output could be used to write the templates, thus improving the automation in DScribe’s approach.

## Discussion of Applications

This section outlines possible applications of wit’s technique that take advantage of its characteristics. wit’s precision is especially handy when generating documentation (discussed in Section 8.1) or tests (Section 8.2). wit’s other key feature (that it’s *lightweight*) helps apply it to different scenarios. For research in mining software repositories, not requiring complete project builds enables scaling analyses to a very large number (e.g., several thousands) of projects—whereas building all of them would be infeasible Hassan et al. (2017). Using wit as a component of a recommender system that runs in real-time is another scenario where speed/scalability would be of the essence.

### Documentation

As we demonstrated in Section 5.6.3, the output of wit’s analysis can be useful to extend, complement, and revise the documentation of public methods’ exceptional behavior. Accurately documenting exceptions is crucial for developers Zhou et al. (2020), but writing documentation is onerous Nassif et al. (2021); Nguyen et al. (2014); as a result, APIs often lack documentation Robillard et al. (2013), especially for exceptions Raymond and Weimer (2008). wit’s high *precision* ensures that its output can generally be trusted without requiring manual validation, and hence it can directly help the job of developers writing documentation (or tests).

In most cases, wit’s exception preconditions are in a form that can be easily transformed into method documentation—for example by expressing them in natural language using pattern matching Zhou et al. (2020); Blasi et al. (2018); Goffi et al. (2016). In fact, since it uses precise static analysis, we found several cases where wit’s exception preconditions provide more rigorous information than what is available in programmer-written documentation. For example, Listing 10 shows the programmer-written exceptional behavior documentation and the initial part of the implementation of a method from class Conversion in project Apache Commons Lang. wit outputs two exception preconditions for the method:

1 $$\begin{aligned} \texttt {src.length > 8} \end{aligned}$$

2 $$ \begin{aligned} \mathtt {src.length <= 8 \& \& srcPos - src.length > -4} \end{aligned}$$

both corresponding to an IllegalArgument exception. At first sight, it may seem that wit’s output is incomplete (it doesn’t mention the preconditions “src is empty” and “src is null” in the Javadoc)^84^ and needlessly verbose (isn’t src.length $\mathtt {<= 8}$ redundant?). A closer look, however, reveals that several aspects of the natural-language documentation are questionable or inconsistent. First, it mixes explicitly and implicitly thrown exceptions: a NullPointer exception is thrown by the Java runtime when evaluating the expression on line 6, not by the method’s implementation. wit ignores such language-level exceptions by design; as we mentioned in Section 3.7, not including implicit exceptions in API documentation may be preferable Forward and Lethbridge (2002); Raymond and Weimer (2008). A second issue with Listing 10’s documentation is that it is incorrect: if src is empty, the method does not throw an IllegalArgument exception; instead, the Java runtime throws an IndexOutOfBounds exception at line 12 (another system-level implicit exception). Finally, Listing 10’s documentation is inconsistent regarding the *order* in which the various exception preconditions are checked: whether src is null is checked first (implicitly), followed by src.length $\mathtt {> 8}$ (explicitly), src.length - srcPos $\mathtt {< 4}$ (explicitly), and whether src is empty (implicitly)—in this order. Thus, predicate src.length $\mathtt {<= 8}$ in wit’s second inferred preconditions is not redundant but rather useful to ensure that the precondition precisely captures the conditions under which a certain path is taken. Admittedly, wit may sometimes present preconditions in a form that is harder to understand for a human; for example, it is questionable that the “simplification” of src.length - srcPos $\mathtt {< 4}$ into srcPos - src.length $\mathtt {> -4}$ improves readability. However, these are just pretty-printing details that are currently left to SymPy; changing them to generate constraints that follow certain preferred templates could be done following Nguyen et al.’s Nguyen et al. (2014) approach. In fact, one could even let the user decide the output format according to their preference. Overall, this example demonstrates that wit’s output often has all the information needed to generate accurate documentation that avoids ambiguities or other inconsistencies.
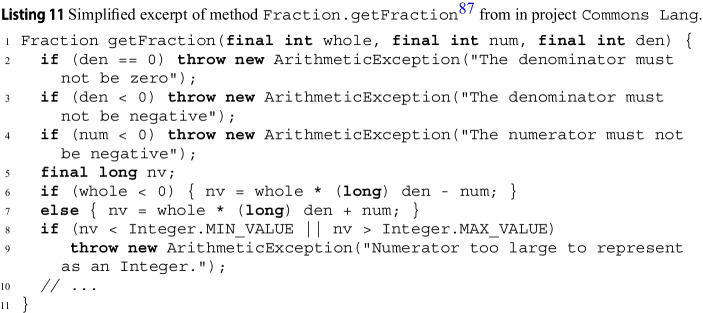


### Generating Tests

Automatically generating tests that exercise^85^ a method’s exceptional behavior is another natural applications of wit. Fully pursuing it is outside this paper’s scope; nevertheless, we briefly discuss this directions on a few concrete examples that we encountered while carrying out Section 4’s empirical evaluation.

As mentioned in Section 3, each exception precondition reported by wit also comes with an example of inputs that satisfy it; for instance, for exception precondition (2), wit outputs the example [src.length=2, srcPos=0]. Writing a test that initializes an array with two elements, calls the method in Listing 10, and checks that an IllegalArgumentException is thrown (and that it contains a specific message) is straightforward. In fact, one could even try to automate the generation of tests and oracles from wit’s examples and preconditions. For example, using property-based testing Claessen and Hughes (2000): after expressing (2) (or even the specific example) as an input property, let a tool like jqwikjqwik: Property-Based Testing in Java: https://jqwik.net/ randomly generate inputs that satisfy it.

The information captured by wit can support increasing the level of automation and generally make programmers more productive. It

can also improve the quality of the tests that are written, as demonstrated by the following example. Listing 11 shows a (simplified) excerpt of method Fraction.getFraction in Apache Commons Lang, which takes three integers whole, num, den, and returns an object representing the fraction $\texttt {whole} + \texttt {num}/\texttt {den}$. As we can see in Listing 11, getFraction has 4 exception preconditions: (a) (line 2) when den is 0; (b) (line 3) when den is negative; (c) (line 4) when num is negative; (d) (line 8) when the resulting numerator nv exceeds the largest integer in absolute value. Commons Lang is a thoroughly tested project Nassif et al. (2021), and in fact all four exceptional behaviors are tested.https://github.com/apache/commons-lang/blob/ce477d9140f1439c44c7a852d7df1e069e21cb85/src/test/java/org/apache/commons/lang3/math/FractionTest.java#L437 The 4 behaviors are not evenly tested though: 3 calls cover (a), 6 calls cover (b) (including three identical calls, which is likely a copy-paste error), 1 call covers (c), and 4 calls cover (d). Comments in the test method which refer to the four categories are sometimes misplaced (for example, two calls under “zero denominator” actually cover (d)). In contrast, wit’s example inputs correspond one-to-one and uniquely to each exception precondition: (a) den=0; (b) den=-1; (c) num=-1, den=1; (d) whole=2147483648, num=0, den=1. If we wanted multiple example inputs for the same precondition, we could just ask Z3 to generate more. In all, wit’s output can be quite useful to guide a systematic test-case generation process.
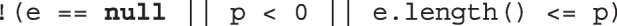


Another situation where wit’s output helps write tests that exercise exceptional behavior is when this requires a combination of inputs for different arguments. One example is Commons Text’s method FormattableUtils.append(),https://github.com/apache/commons-text/blob/04748ac3693163685e411167e5c689eb9ae98dac/src/main/java/org/apache/commons/text/FormattableUtils.java#L90 which takes 6 arguments and comes from Java’s Formatter interface.https://docs.oracle.com/en/java/javase/18/docs/api/index.htmlFormattableUtils.append()’s exception precondition involves the negation of a disjunction of three Boolean predicates: 
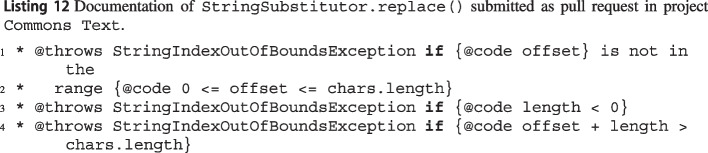
. wit suggests an example input where e.length() is 1, and p is 0, which is easy to implement as a test. Another example is method StringSubstitutor.replace()https://github.com/apache/commons-text/blob/04748ac3693163685e411167e5c689eb9ae98dac/src/main/java/org/apache/commons/text/StringSubstitutor.java#L742 in the same project, which takes three arguments (one character array and two integers) and may throw an exception in a nested call. As regularly seen in Apache Commons projects, the method accepts null or empty arrays; however, when the array is non-null, the exception precondition gets quite complex. wit provides exception triggering inputs for the three arguments, including that the character array must not be null and could be empty. In cases like this, we could reuse parts of wit’s extracted precondition to document the complex exception condition. The complexity of the precondition, together with it being in a nested call, may be the reason why the documentation and tests were missing in the project.

## Conclusions

We presented wit: a static analysis technique to extract exception preconditions of Java methods. wit focuses on precision: it only reports correct preconditions.

An evaluation on 46 open-source Java libraries and five JDK 11 modules demonstrated also that it is lightweight (under two seconds per analyzed public method on average), precise (all inferred preconditions are correct), and can recover a significant fraction of the known exception preconditions (9–83% of the supported exception preconditions using Nassif et al. (2021)’s manual analysis as ground truth).

While the exception preconditions detected by wit tend to be syntactically simple, they often complement the available documentation of a method’s exceptional behavior, as we demonstrated by merging a selection of 170 inferred exception precondition as pull requests in the projects’ open source repositories.

In order to combine scalability and applicability, wit can perform a modular analysis: after inferring the exception preconditions of a project A, it can use them to analyze the behavior of another project B whenever it calls out to any methods in A. Our empirical analysis suggested that modular analysis is a bit of a mixed bag: it does increase the number of exception precondition wit can detect, but it may also decrease the precision for the so-called “maybes”—exception preconditions that are reported separately, as wit could not conclusively establish that they are correct. Accordingly, wit can be configured to use modular analysis selectively, according to what is the main goal of its users. Investigating heuristics to help the automatic selection of these configuration options is an interesting direction for future work.

## Data Availability

The artifacts that support the findings of this study are available in https://doi.org/10.6084/m9.figshare.22217014.

## Footnotes

1 The range depends on which features and which output of wit we consider; see Section 5.2 for all details.

2 wit replication package: https://doi.org/10.6084/m9.figshare.22217014

3 Apache Dubbo https://dubbo.apache.org/en/

4 Apache Commons Lang https://commons.apache.org/proper/commons-lang/

5 Apache Dubbo on GitHub https://github.com/apache/dubbo

6 https://github.com/apache/commons-lang/commit/ba607f525b842661d40195d0d4778528e2384e70

7 JavaParser: https://github.com/javaparser/javaparser

8 JGraphT: https://jgrapht.org/

9 https://commons.apache.org/proper/commons-lang/apidocs/org/apache/commons/lang3/ArrayUtils.html

10 A simple path is one where any one node appears at most once. We compute them using JGraphT’s JAllDirectedPaths method.

11 https://jgrapht.org/javadoc-1.4.0/org/jgrapht/alg/shortestpath/AllDirectedPaths.html

12 “Modular analysis” simply refers to wit’s capability of reusing the exception preconditions of previously analyzed projects. The user controls how this capability is applied: wit will always access the complete source code of the project or projects given to it as input; if the user wants to analyze a project *B* separately from another project *A*, they will have to run wit twice (once on *B*, and then once on *A*) with modular analysis enabled.

13 “Expres” and “maybes” are precisely introduced in Section 3.5. In a nutshell, expres come from expaths that are provably feasible, and hence they are correct by constructions; maybes come from expaths with inconclusive feasibility analysis, and hence they are just educated guesses that may be incorrect.

14 https://github.com/apache/commons-text/blob/21fc34f17175aba66f55fb6f805e60c13055da49/src/main/java/org/apache/commons/text/RandomStringGenerator.java#L362-L366

15 https://github.com/apache/commons-lang/blob/ce477d9140f1439c44c7a852d7df1e069e21cb85/src/main/java/org/apache/commons/lang3/Validate.java#L107-L111

16 https://www.mongodb.com/

17 https://github.com/javaparser/javaparser/tree/master/javaparser-core-serialization/src/main/java/com/github/javaparser/serialization

18 https://github.com/square/moshi

19 wit’s Z3 ad hoc encoding also handles aliasing by explicitly keeping track of possible aliases along each checked path. Thanks to the other heuristics that limit path length (Section 3.7), this approach is feasible in practice.

20 SymPy https://www.sympy.org/en/index.html

21 As a simple example, as done for testing Fraser and Arcuri (2015), before every array access such as  add a guard , so that the implicitly thrown exception becomes explicit.

22 Even mature static analysis frameworks such as Spoon have only partial/experimental support for features such as try/catch.

23 https://github.com/INRIA/spoon/tree/6d157f35491eabe6e7f7505a8ebc22a9694f491f/spoon-control-flow

24 https://github.com/openjdk/jdk/tree/jdk-11%2B28

25 We exclude 6 innacurate cases.

26 To ensure a more varied sample, we targeted $3+3$ methods that use the JDK and $3 + 3$ that do not.

27 https://github.com/apache/accumulo/pull/2594

28 https://github.com/apache/commons-lang/pull/869

29 https://github.com/apache/commons-lang/pull/870

30 https://github.com/apache/commons-lang/pull/871

31 https://github.com/apache/commons-math/pull/206

32 https://github.com/apache/commons-math/pull/207

33 https://github.com/apache/commons-text/pull/311

34 https://github.com/apache/commons-io/pull/339

35 We pick all expres for 7 projects with less than 10 expres in total.

36 To keep the manual analysis manageable, this sample size (218) is sufficient to estimate the precision of maybes with up to 5% error and 95% probability but with a stronger (i.e., 83%) a priori assumption.

37 Excluding unsupported annotation kinds is a common practice in the empirical evaluation of tools that infer annotations Zhou et al. (2020).

38 https://github.com/apache/commons-io/blob/2ae025fe5c4a7d2046c53072b0898e37a079fe62/src/main/java/org/apache/commons/io/EndianUtils.java

39 The dataset contains 503 parameter rules for 9 projects; we manually analyzed 175 from projects asm, Commons IO, and jfreechart.

40 https://asm.ow2.io/asm4-guide.pdf#page=62

41 https://github.com/jfree/jfreechart

42 A subset of the 742 expres we checked for correctness in Section 5.1.

43 A common instance of this programming pattern occurs when implementing immutable data structures. For example, state-modifying List interface methods such as add in class UnmodifiableList, which is instantiated by method unmodifiableList in java.util.Collections.

44 https://github.com/openjdk/jdk/blob/da75f3c4ad5bdf25167a3ed80e51f567ab3dbd01/src/java.base/share/classes/java/util/Collections.java#L1312

45 https://github.com/apache/lucene-solr/blob/7ada4032180b516548fc0263f42da6a7a917f92b/solr/solrj/src/java/org/apache/solr/client/solrj/io/sql/ResultSetImpl.java#L631

46 https://github.com/apache/logging-log4j2/blob/59f6848b70eebbaa3aa0e14f7186b9b5e1942b5a/log4j-layout-template-json/src/main/java/org/apache/logging/log4j/layout/template/json/util/TruncatingBufferedWriter.java#L160

47 https://github.com/apache/logging-log4j2/blob/59f6848b70eebbaa3aa0e14f7186b9b5e1942b5a/log4j-perf/src/main/java/org/apache/logging/log4j/perf/nogc/OpenHashStringMap.java#L476

48 https://github.com/apache/jackrabbit/blob/35d5732bc1418718f49553a81e42ac4146619dcf/jackrabbit-spi-commons/src/main/java/org/apache/jackrabbit/spi/commons/name/PathFactoryImpl.java#L217

49 https://github.com/apache/commons-lang/blob/5def1c8d634f12a265662f38188cd611aa1e574b/src/main/java/org/apache/commons/lang3/ArrayUtils.java#L2807

50 https://github.com/openjdk/jdk/blob/da75f3c4ad5bdf25167a3ed80e51f567ab3dbd01/src/java.base/share/classes/java/lang/String.java#L1002

51 https://github.com/openjdk/jdk/blob/da75f3c4ad5bdf25167a3ed80e51f567ab3dbd01/src/java.base/share/classes/java/lang/String.java#L142-L153

52 https://openjdk.org/jeps/254

53 https://github.com/openjdk/jdk/blob/da75f3c4ad5bdf25167a3ed80e51f567ab3dbd01/src/java.base/share/classes/java/lang/String.java#L140

54 https://github.com/apache/pdfbox/blob/2fcdf26e400952357bef4276121bd59fb7e4040a/pdfbox/src/main/java/org/apache/pdfbox/pdmodel/interactive/annotation/PDAnnotationWidget.java#L110

55 https://github.com/openjdk/jdk/blob/da75f3c4ad5bdf25167a3ed80e51f567ab3dbd01/src/java.base/share/classes/java/lang/Double.java#L555

56 https://github.com/openjdk/jdk/blob/da75f3c4ad5bdf25167a3ed80e51f567ab3dbd01/src/java.base/share/classes/java/lang/Double.java#L50-L62

57 https://github.com/openjdk/jdk/blob/da75f3c4ad5bdf25167a3ed80e51f567ab3dbd01/src/java.base/share/classes/java/util/Stack.java#L80

58 Remember that wit targets only top-level public methods, but may follow paths that go into private members.

59 https://github.com/openjdk/jdk/blob/da75f3c4ad5bdf25167a3ed80e51f567ab3dbd01/src/java.base/share/classes/java/util/Vector.java#L112

60 https://github.com/apache/pdfbox/blob/2fcdf26e400952357bef4276121bd59fb7e4040a/pdfbox/src/main/java/org/apache/pdfbox/pdmodel/common/function/type4/ExecutionContext.java#L65

61 https://github.com/apache/commons-text/blob/21fc34f17175aba66f55fb6f805e60c13055da49/src/main/java/org/apache/commons/text/similarity/IntersectionResult.java#L58

62 https://github.com/openjdk/jdk/blob/da75f3c4ad5bdf25167a3ed80e51f567ab3dbd01/src/java.base/share/classes/jdk/internal/org/objectweb/asm/util/ASMifier.java#L1120

63 https://github.com/apache/curator/blob/4a11aaef8b190dc220d35b7a91df294bfa06250e/curator-client/src/main/java/org/apache/curator/utils/PathUtils.java#L46

64 https://github.com/apache/commons-io/blob/2ae025fe5c4a7d2046c53072b0898e37a079fe62/src/main/java/org/apache/commons/io/FileUtils.java#L1482-L1483

65 https://github.com/openjdk/jdk/blob/da75f3c4ad5bdf25167a3ed80e51f567ab3dbd01/src/java.base/share/classes/java/net/URLConnection.java#L390

66 https://github.com/openjdk/jdk/blob/da75f3c4ad5bdf25167a3ed80e51f567ab3dbd01/src/java.base/share/classes/java/net/URLConnection.java#L433

67 https://github.com/openjdk/jdk/blob/da75f3c4ad5bdf25167a3ed80e51f567ab3dbd01/src/java.base/share/classes/java/util/zip/Deflater.java#L567-L573

68 https://github.com/openjdk/jdk/blob/da75f3c4ad5bdf25167a3ed80e51f567ab3dbd01/src/java.base/share/classes/jdk/internal/reflect/UnsafeStaticShortFieldAccessorImpl.java#L39-L41

69 https://github.com/openjdk/jdk/blob/da75f3c4ad5bdf25167a3ed80e51f567ab3dbd01/src/java.base/share/classes/jdk/internal/reflect/UnsafeStaticLongFieldAccessorImpl.java#L104

70 https://docs.oracle.com/en/java/javase/11/docs/api/java.base/java/time/package-summary.html

71 https://github.com/openjdk/jdk/blob/da75f3c4ad5bdf25167a3ed80e51f567ab3dbd01/src/java.base/share/classes/java/time/Period.java#L321

72 https://github.com/openjdk/jdk/blob/339ca887835d6456da9fcccdc32fb7716cbc60bb/src/java.base/share/classes/java/io/StringBufferInputStream.java#L113

73 https://github.com/openjdk/jdk/blob/339ca887835d6456da9fcccdc32fb7716cbc60bb/src/java.base/share/classes/java/io/Reader.java#L168

74 https://github.com/openjdk/jdk/blob/da75f3c4ad5bdf25167a3ed80e51f567ab3dbd01/src/java.base/share/classes/java/lang/Integer.java#L614

75 https://github.com/openjdk/jdk/commit/564011cff0667c6d34cf6aa46eedd11f2e01862b

76 https://github.com/apache/commons-lang/commit/656d2023dcd149018cd126e283f675b4ffef9715

77 https://github.com/apache/commons-io/commit/ff387016c2d95162aa6bf6735be47c559751b530

78 https://blog.joda.org/2012/11/javadoc-coding-standards.html

79 https://bytebuddy.net/#/develop

80 https://dubbo.apache.org/en/docs/v2.7/dev/checklist/

81 https://issues.apache.org/jira/browse/LANG-1681

82 https://github.com/apache/commons-lang/pull/1047

83 One maintainer from Accumulo remarked that ours “are nice fixes to the javadoc, thanks for finding them.”

84 https://github.com/apache/commons-lang/blob/ce477d9140f1439c44c7a852d7df1e069e21cb85/src/main/java/org/apache/commons/lang3/Conversion.java#L373

85 https://github.com/apache/commons-lang/blob/ce477d9140f1439c44c7a852d7df1e069e21cb85/src/main/java/org/apache/commons/lang3/math/Fraction.java#L171
